# Spatial and Temporal Distribution of Soil-Applied Neonicotinoids in Citrus Tree Foliage

**DOI:** 10.1093/jee/toy114

**Published:** 2018-04-23

**Authors:** Kevin W Langdon, Rhonda Schumann, Lukasz L Stelinski, Michael E Rogers

**Affiliations:** Department of Entomology and Nematology, Citrus Research and Education Center, University of Florida, Lake Alfred, FL, USA

**Keywords:** neonicotinoid, citrus, *Diaphorina citri*, soil application, citrus greening

## Abstract

*Diaphorina citri* Kuwayama (Hemiptera: Liviidae) is the insect vector of *Candidatus* Liberibacter asiaticus (*C*Las), the presumed cause of huanglongbing (HLB) in citrus (Rutaceae). Soil-applied neonicotinoids are used to manage vector populations and thus reduce the spread of HLB in Florida citrus. Studies were conducted in the greenhouse and field to quantify the spatial and temporal distribution of three neonicotinoid insecticides within individually sampled leaves and throughout the tree canopy. Following field application, no difference in parent material titer was observed between leaf middles versus leaf margins following application of Platinum 75SG or Belay 2.13SC; however, imidacloprid titer was higher in leaf margins than leaf middle following application of Admire Pro. The bottom region of trees contained more imidacloprid than other regions, but was not different from the spherical center region. In the greenhouse, imidacloprid and clothianidin titers peaked 5 wk following application of Admire and Belay, respectively, and thiamethoxam titer peaked 3 wk after application of Platinum. There was no effect of leaf age on uptakes of any insecticides tested. Titers of soil-applied neonicotinoids quantified in the field failed to reach known levels required to kill *D. citri*. Exposure of *D. citri* to sublethal dosages of neonicotinoids is of concern for HLB management because of possible failure to protect treated plants from *D. citri* and selection pressure for development of neonicotinoid resistance. Our results suggest that current soil-based use patterns of neonicotinoids for *D. citri* management may be suboptimal and require reevaluation to maintain the utility of this chemical class in citrus.

The Florida citrus (Rutaceae) industry has severely declined over the last decade, due to the combined introductions of the Asian citrus psyllid, *Diaphorina citri* Kuwayama (Hemiptera: Liviidae), and the presumed causal agent of citrus greening disease, *Candidatus* Liberibacter asiaticus (*C*Las) (Halbert and Manjunath 2004, Bové 2006). Citrus greening disease, or huanglongbing (HLB), was first detected in the state in 2005, 7 yr after the discovery of the insect vector, *D. citri* (Halbert and Manjunath 2004). The citrus industry in Florida was valued at US$9.9 billion during 2014 and 2015 and is the single largest agricultural commodity in the state (Hodges and Spreen 2015). Following innoculation by *C*Las-positive *D. citri*, bacteria move from the infection site through the phloem, eventually accumulating in the roots (Trivedi et al. 2012). As disease symptoms begin to manifest, the root system declines (Johnson et al. 2014). In turn, the tree canopy is starved for nutrients, causing leaf and fruit drop, thereby reducing yield in the near term, and eventually resulting in tree death (Halbert and Manjunath 2004, Bové 2006, Grafton-Cardwell et al. 2013). Various methods of HLB management have been investigated, including repeated releases of the biological control agent, *Tamarixia radiata* Waterston (Hymenoptera: Eulophidae), nursery sanitation, and roguing of infected trees in the field, among other strategies (Stansly and Rogers 2006, Hall and Albrigo 2007, Hall et al. 2008). Given existing disease impact and an estimated infection rate of *D. citri* in the state between 80 and 100% (Coy and Stelinski 2015), insecticides have become the primary tool used in an effort to slow disease spread, with emphasis on soil-applied neonicotinoids in young tree plantings (Rogers 2008, 2013). Young trees do not bear fruit and are typically categorized as those less than 8 feet in height (Hall and Albrigo 2007, Rogers 2012). Unlike mature citrus trees, nonbearing trees produce vegetative flush often throughout the year, which places them at great risk to *C*Las infection (Stansly and Rogers 2006).

*D. citri* adults are attracted to volatiles emitted by actively growing flush shoots (Patt and Sétamou 2010), which is the only resource for oviposition and nymph development (Tsai and Liu 2000). Newly hatched nymphs feed on phloem sap of the developing flush shoots where nymphs acquire the bacterium from infected plants (Pelz-Stelinski et al. 2010). As newly infected nymphs reach adulthood, they disperse and subsequently inoculate other uninfected trees. In an attempt to break this cycle, Rogers (2008) developed a program of rotating between neonicotinoids applied to the soil with foliar sprays of alternate modes of action. Neonicotinoids are highly systemic, xylem-mobile insecticides within the Insecticide Resistance Action Committee (IRAC) subgroup 4A and are often applied to the soil for transport to the plant foliage (Elbert et al. 2008). Three neonicotinoid insecticides are labeled for use in nonbearing citrus in Florida: thiamethoxam (Platinum 75 SG—Syngenta Crop Protection, Inc., Greensboro, NC), imidacloprid (Admire Pro 4.6F—Bayer CropScience, Research Triangle Park, NC), and clothianidin (Belay 2.13 SC—Valent USA Corporation, Walnut Creek, CA). Previous investigations documented residual *D. citri* adult and/or nymph control of 6–11 wk after neonicotinoids were applied to the soil (Qureshi and Stansly 2007, 2009; Ichinose et al. 2010; Sétamou et al. 2010; Rogers 2012; Byrne et al. 2017). However, even in the most intensively managed citrus groves, HLB infection rates in commercial Florida citrus groves continued to increase at an estimated rate of 1–3% annually (Rogers 2013).

Little is known regarding the movement and distribution of soil-applied neonicotinoids through citrus tissues. Boina et al. (2009) proposed that uneven temporal and spatial distribution in citrus tissue may cause exposure of *D. citri* to sublethal doses of insecticide. Furthermore, uneven uptake of systemic insecticides by the root system makes it possible for *D. citri* to develop (Rogers 2012). Previous studies that quantified neonicotinoid concentration in citrus sampled either xylem fluid or entire leaves and quantified parent material concentrations using enzyme-linked immunosorbent assay (ELISA) (Castle et al. 2005, Garlapati 2009, Sétamou et al. 2010). When quantifying chemical constituents using ELISA, one cannot differentiate between parent material and resulting metabolites. Nevertheless, Castle et al. (2005) found no difference in the spatial distribution of imidacloprid or thiamethoxam throughout citrus tree canopy xylem fluid for control of the glassy-winged sharpshooter, *Homalodisca coagulata* (Say) (Hemiptera: Cicadellidae). They sampled xylem fluid from branch shoots, which is not consistent with the phloem-feeding patterns of *D. citri*. In addition, the authors applied insecticides through a microirrigation system, which evenly distributes insecticide around the tree trunk at the time of application. In contrast, Florida growers typically use a drench application device mounted to a four-wheel utility vehicle to apply a solution of approximately 237 ml (water + insecticide) to the soil below only one side of each tree (Rogers M., personal observation). This application method may result in an uneven distribution of insecticide within a tree canopy. In the case of Florida citrus, quantifying the spatial distribution of neonicotinoids within a tree, as well as within a single leaf, is essential to understanding the potential dosages that *D. citri* receive.

Sétamou et al. (2010) correlated percentage control of *D. citri* nymphs with imidacloprid concentration in citrus leaf tissues. They determined that between 200 and 250 parts per billion (ppb) was required to provide control of *D. citri* nymphs in citrus. Because practically all *D. citri* adults are infected with *C*Las in Florida, the concentration required to provide control of adult *D. citri* is likely of greater significance, given that interruption of inoculation will help prevent spread of HLB. A series of more recent studies found that 62.19 parts per million (ppm) of imidacloprid was required to kill 90% of a laboratory *D. citri* adult population when administered by ingestion (Langdon and Rogers 2017). Langdon and Rogers (2017) suggested that feeding deterrence of *D. citri* at sublethal dosages may have contributed to the control associated with neonicotinoid application reported by Sétamou et al. (2010). In addition, Langdon et al. (2018) determined that 64.63 ppm of thiamethoxam was required to achieve a 1% probability of encountering a flush shoot with at least one adult *D. citri* in the field, whereas only 19.05 ppm of thiamethoxam was required to achieve a 1% probability of encountering a flush shoot with at least one *D. citri* nymph in the field, as determined by liquid chromatography-mass spectrometry (LC-MS). This suggests that nymphs are more susceptible to neonicotinoids than adults. The highest mean thiamethoxam titer observed in their field study was 33.39 ppm, more than 30 ppm below the threshold for a tree predicted to be free of *D. citri* adults. The magnitude of difference between dosages required to achieve high mortality levels of *D. citri* or perceived control and the actual titer of thiamethoxam measured within treated trees may partially explain why HLB infection incidence continues to rise despite intense use of soil-applied neonicotinoids in the field.

Although spread of HLB is of great concern, sublethal dosages resulting from uneven spatial and temporal distribution may also increase selection of resistance to neonicotinoids within populations of *D. citri*. Tiwari et al. (2011) documented resistance to neonicotinoids in the field in 2009, but no resistance was detected in subsequent studies conducted in 2014 (Coy et al. 2016). This shift was thought to be due to the implementation of area-wide spray programs that rotated non-neonicotinoid insecticides over broad acreages in a coordinated fashion (Rogers et al. 2012). However, resistance to imidacloprid was again detected in 2016 in isolated field populations of *D. citri* throughout the state (Langdon and Rogers 2017).

Quantifying uptake and distribution patterns of neonicotinoids in citrus leaf tissues following soil application would improve understanding of *D. citri* management in Florida citrus. In addition, quantifying exposure of *D. citri* to sublethal insecticide dosages may help efforts to prevent resistance to neonicotinoids. The purpose of this study was to quantify the spatial and temporal distribution of all analytes resulting from the soil application of three neonicotinoid insecticides in citrus leaf tissues using an application method commonly implemented by Florida citrus growers.

## Materials and Methods

### Spatial and Temporal Neonicotinoid Distribution

#### Greenhouse Study

A greenhouse study was conducted to evaluate the uptake of three neonicotinoid insecticides following application to the soil. The distribution of insecticide residue within citrus leaves was evaluated. Small citrus trees (ca. 0.08m^3^ canopy volume) were planted to 11.4-liter pots containing a blend of 50% sand and 50% potting media (Sun Gro Horticulture, Fafard Professional Potting Mix). Plots consisting of four trees were arranged in a randomized complete block design (RCBD) with four treatments and four replicates. Treatments consisted of an untreated control, Platinum 75SG, Admire Pro 4.6F, and Belay 2.13SC applied at the recommended rate for nonbearing citrus trees based on 346 trees per hectare (140 trees per acre) (Table 1). A single insecticide application was made by applying 237 ml of insecticide solution (deionized water + insecticide) into each pot. Leaf tissue samples were randomly collected prior to the application of insecticides and then weekly for 13 wk following the application. At each sample date, four leaves across each of the four trees within a plot were harvested. Each leaf was excised into two sections: 1) Middle (area inclusive of 0.5 cm on either side of the mid-vein extending from leaf petiole to 0.5 cm from leaf tip) and 2) Margin (remainder of leaf not associated with the ‘middle’ leaf section). Leaf material from each section within a plot was wrapped separately in a labeled heavy duty aluminum foil and collectively stored by treatment in a plastic resealable bag at −20°C until residue analyses were conducted.

**Table 1.** Neonicotinoid product description and use rates for greenhouse and field studies

Product	Rate per hectare applied (rate per acre)	Rate per tree (based on 346 trees per hectare or 140 trees per acre)	Grams active ingredient per tree	Resulting analytes
Admire Pro 4.6F	511.09 ml/ha (7 fl oz/ac)	1.48 ml/tree	0.814 g/tree	imidacloprid*
				5-OH
				Olefin
Platinum 75SG	128.10 g/ha (1.83 oz wt/ac)	0.37 g/tree	0.324 g/tree	thiamethoxam*
				Clothianidin
				TZMU
				TZNG
Belay 2.13SC	438.07 ml/ha (6 fl oz/ac)	1.27 ml/tree	0.278 g/tree	clothianidin*
				TZMU
				TZNG

*Active ingredient of listed formulated product.

#### Two-Season by Two-Location Field Study

A field study was conducted at two commercial grove locations across two seasons to evaluate the spatial and temporal distribution of three neonicotinoid insecticides in citrus trees following application to the soil. Nonbearing (v. Hamlin/r.s. Swingle) trees of similar size and age (ca. 1.3m^3^ canopy volume and field planted approximately 18 mo prior to the first application) were identified in two commercial groves, each of which represent a major citrus production area of Florida (pine ‘flatwoods’, 26.6329, −81.5389 and ‘central ridge’, 27.2765, −81.3802). The low lying flatwoods location was a continuous solid-set planting of trees of the same age. The trees were planted to sandy soils comprised of 96.4% sand, 2% clay, and 1.6% silt with 0.68% organic matter and cation exchange capacity (CEC) of 14.2 meq/100 g. The central ridge location contained a mature grove with randomly interspersed younger trees that had been replanted. This grove was comprised of sandy soils with 98.4% sand, 1.6% clay, and 0% silt with 0.59% organic matter and CEC of 4.1 meq/100 g. The central ridge site was centrally located on the North–South ridge running through central Florida spanning from near Orlando to south of Lake Placid. Both groves had irrigation installed, which delivered water evenly within the canopy drip line. At each site, plots were arranged in a RCBD with four treatments and four replicates. Treatments consisted of an untreated control, Platinum 75SG, Admire Pro 4.6F, and Belay 2.13SC applied at the recommended rate for nonbearing citrus trees based on 346 trees per hectare (140 trees per acre) (Table 1). At each location, the first season insecticide application was made on 19 August 2015 and the second season application was made on 13 January 2016. At the time of application, 237 ml of insecticide solution (deionized water + insecticide) was applied to the soil at the base of each tree trunk. At the flatwoods location, tree rows were oriented north–south, and the application was made on the west side of the tree trunk. At the central ridge location, tree rows were oriented east–west, and the application was made on the south side of the tree trunk. Leaf tissue samples were collected prior to the application of insecticides and then weekly for 12 wk following the application. Trees were divided into seven tree regions: bottom (lower 10% of canopy), spherical center, top (upper 10% of canopy), and four cardinal sides (east, west, south, and north), sampled near the equatorial circumference of the canopy. At each sample date, four leaves from each of the seven tree regions across each of the four trees within a plot were harvested. Each leaf was excised into two sections: 1) Middle (area inclusive of 0.5 cm on either side of the mid-vein extending from leaf petiole to 0.5 cm from leaf tip), and 2) Margin (remainder of leaf not associated with the ‘middle’ leaf section). Leaf material from each leaf section and each tree region within a plot was wrapped individually in a labeled heavy duty aluminum foil and stored collectively in a resealable plastic bag at −20°C until residue analyses were conducted. To evaluate distribution of analytes within a leaf, only leaf tissues from the ‘top’ tree region were used to confirm within-leaf residue distribution observed in the greenhouse. To evaluate temporal expression differences and to determine the distribution of analytes throughout the tree canopy, only leaf tissues from the ‘middle’ leaf section were used.

### Effect of Leaf Maturity on Neonicotinoid Expression

#### Two-Season Field Study

A field study was conducted across two seasons to determine the effect of leaf maturity on expression of each of three neonicotinoids following application to the soil. Untreated, nonbearing citrus trees (v. Hamlin/r.s. Swingle) (ca. 1.5m^3^ canopy volume) in a research grove (27.7279, −80.4564) were used in the study. Trees were field planted approximately 22 mo prior to the first insecticide application to sandy soil comprised of 96.8% sand, 1.6% silt, and 2% clay, with 1.04% organic matter and CEC of 6.7 meq/100 g. Trees were planted using a 2.4-m in-row spacing and 2.4-m between-row spacing, which provided sufficient separation to eliminate uptake of insecticides applied to an adjacent tree, confirmed by analysis of trees in the untreated control. The study was arranged in a RCBD with four treatments and four replicates. Treatments consisted of an untreated control, Platinum 75SG, Admire Pro 4.6F, and Belay 2.13SC applied at the recommended rate for nonbearing citrus trees based on 346 trees per hectare (140 trees per acre) (Table 1). Approximately 14 d prior to each insecticide application, a gas-powered hedge trimmer was used to trim the tree canopy to a mean canopy volume (MCV) of approximately 1.3 m^3^ to promote flushing. The first season insecticide application was made on 5 May 2017 and the second season application was made on 21 June 2017, each when flush shoots were approximately 2.5 cm long. At the time of application, 237 ml of insecticide solution (deionized water + insecticide) was applied to the soil at the base of each tree trunk. Leaf tissue samples were collected prior to the application of insecticides and then weekly for 4 wk following the application. At each sample date, four mature leaves and four flush shoots were harvested across each of the four trees within a plot; one mature leaf and one flush shoot were pulled from each cardinal region within each tree. Mature leaves and flush shoots were placed into separate labeled paper bags and collectively stored by treatment in a plastic resealable bag at −20°C until residue analyses were conducted. The same cohort of flush shoots was sampled each week to control for potential differences in expression values in flush that had not yet formed at the time of application in later sampling dates.

### Extraction and Leaf Tissue Analysis

The extraction and leaf tissue analysis methodology was described in detail elsewhere (Langdon et al. 2018). In brief, leaf material from each plot was ground to a fine powder using liquid nitrogen and mortar and pestle. A ca. 5-g subsample of leaf powder was weighed and transferred to a 20-ml glass vial with a Teflon-lined cap and stored at −20°C until extraction; the exact weight of each sample was recorded for conversion of analyte concentration to the fresh leaf weight basis. Extraction was conducted using QuEChERS in 15-ml acetonitrile using preweighed reagent sachets (United Chemical Technologies, no. ECQUEU7-MP). A cleanup step was then conducted in which chlorophyll was removed from the acetonitrile extract using ChloroFiltr polymeric–based sorbent tubes (United Chemical Technologies, no. ECMPSGG15CT). The supernatant from cleanup was then filtered through a 20-µm Teflon filter into an auto-sampler vial. Separation and quantification of analytes were accomplished using ultra-high performance liquid chromatography with a C-18 column coupled to a Thermo TSQ Quantum mass spectrometer. The aqueous mobile phase was 0.1% formic acid in water and the polar modifying phase was 0.1% formic acid in acetonitrile. Samples were run against standards to construct a five-point linear curve in a concentration range of 0.5–50 ppm, and then against a five-point standard curve in the range of 5–300 ppb. The concentration represented by the curve (in extract solution) was then converted back to µg/g leaf tissue using the exact sample weight.

### Statistical Analyses

#### In-Leaf Distribution of Neonicotinoids

Chemical titer data for greenhouse leaf section means were averaged over replicate and subjected to a general linear-mixed model to test for sample date by leaf section interactions. For leaf section field data, only chemical titer leaf section means from the ‘top’ tree region were averaged over replicate and subjected to a general linear mixed model to test for sample date by leaf section interactions; location was treated as a random effect.

#### Temporal Expression of Neonicotinoids

Chemical titer data for greenhouse means were averaged over replicate and subjected to a general linear mixed model to test for sample date by leaf section interactions. For field data, chemical titer means from only the ‘middle’ leaf section were averaged over replicate and subjected to a general linear mixed model to test for sample date by location and sample date by tree region interactions.

#### Spatial Distribution of Admire Pro Analytes Throughout the Tree Canopy

Chemical titer data were averaged over replicate and subjected to a general linear-mixed model to test for location by tree region and sample date by tree region interactions.

#### Effect of Leaf Maturity on Neonicotinoid Expression

Chemical titer data were averaged over replicate and subjected to a general linear mixed model to test for sample date by leaf maturity interactions; season was treated as a random effect.

In all cases, means were square-root transformed prior to analysis to achieve homogeneity of variance meeting the assumptions of the model. Each of the four models above was adjusted for cumulative rainfall. Data were combined across years or seasons for analysis. Analyses were performed with SASv9.4 (Proc GLIMMIX, SAS Institute, 2013). Mean separations indicate differences between tree regions at α ≤ 0.05.

## Results

### In-Leaf Distribution of Neonicotinoids

Following application of Admire Pro in the greenhouse, no sample date by leaf section interaction was observed for imidacloprid (*F*_11,1_ = 2.14; *P* = 0.4914), 5-OH (*F*_12,4.107_ = 1.2; *P* = 0.4682), or olefin (*F*_12,18.4_ = 2.42; *P* = 0.0532). Furthermore, no significant difference in titer was observed between leaf margin and leaf center for imidacloprid (*F*_1,8.654_ = 1.97; *P* = 0.1950; Table 2), 5-OH (*F*_1,7.393_ = 1.82; *P* = 0.2175; Table 2), or olefin (*F*_1,12.57_ = 1.37; *P* = 0.2643; Table 2).

**Table 2.** Chemical titer (ppm) in citrus leaf tissue across two leaf sections following application of Admire Pro (1.48 ml per tree) to the soil in the greenhouse and in the field

Study	Leaf section	Imidacloprid		5-OH		Olefin	
		Mean	95% CI	Mean	95% CI	Mean	95% CI
Greenhouse	Center	109.930a	(44.819–175.041)	15.399a	(8.777–22.021)	3.571a	(1.738–5.404)
	Margin	129.310a	(64.199–194.421)	21.217a	(14.595–27.839)	4.959a	(3.126–6.791)
		*P*-value = 0.1950		*P*-value = 0.2175		*P*-value = 0.2643	
Field	Center	0.412b	(0.295–0.528)	0.078b	(0.062–0.095)	0.015a	(0.009–0.022)
	Margin	0.528a	(0.406–0.650)	0.110a	(0.092–0.127)	0.019a	(0.012–0.026)
		*P*-value = 0.0415		*P*-value = 0.0005		*P*-value = 0.2699	

Values sharing the same letter do not differ significantly at α ≤ 0.05.

When Admire Pro was applied to the soil in the field, no sample date by leaf section interaction was observed for imidacloprid (*F*_9,276_ = 0.19; *P* = 0.9948), 5-OH (*F*_9,275.9_ = 0.27; *P* = 0.9832), or olefin (*F*_9,275.9_ = 0.64; *P* = 0.7631). A significant difference in titer between leaf sections was observed for imidacloprid (*F*_1,276_ = 4.19; *P* = 0.0415; Table 2) and 5-OH (*F*_1,275.9_ = 12.27; *P* = 0.0005; Table 2) where the leaf margins contained higher concentrations than the leaf centers. No difference in titer was observed between leaf sections for olefin (*F*_1,275.9_ = 1.22; *P* = 0.2699; Table 2).

Following the application of Platinum 75SG in the greenhouse, no sample date by leaf section interaction was observed for thiamethoxam (*F*_12,1_ = 1.38; *P* = 0.5894), clothianidin (*F*_12,1_ = 6.04; *P* = 0.3088), or TZMU (*F*_12,27.2_ = 0.94; *P* = 0.5267). No significant difference was observed in titer between leaf margin and leaf center for thiamethoxam (*F*_1,23.67_ = 1.05; *P* = 0.3158; Table 3) or for clothianidin (*F*_1,2.981_ = 4.11; *P* = 0.1363; Table 3); however, the leaf margin contained more TZMU than the leaf center (*F*_1,17.12_ = 12.44; *P* = 0.0026; Table 3). A sample date by leaf interaction was observed for TZNG (*F*_12,14.4_ = 4.52; *P* = 0.0042), but the order between leaf sections remained constant over time with the exception of 11 wk following application. Nevertheless, no significant difference was observed in titer between leaf margin and leaf center for TZNG (*F*_1,12.83_ = 4.41; *P* = 0.0561).

**Table 3.** Chemical titer (ppm) in citrus leaf tissue across two leaf sections following application of Platinum 75SG (0.37g per tree) to the soil in the greenhouse and in the field

Study	Leaf section	Thiamethoxam		Clothianidin		TZMU		TZNG	
		Mean	95% CI	Mean	95% CI	Mean	95% CI	Mean	95% CI
Greenhouse	Center	94.162a	(54.080–134.244)	45.201a	(29.866–60.536)	0.796a	(0.081–1.512)	3.637a	(2.875–4.399)
	margin	104.660a	(64.578–144.742)	54.230a	(38.895–69.565)	1.052b	(0.340–1.765)	4.776a	(4.014–5.538)
		*P*-value = 0.3158		*P*-value = 0.1363		*P*-value = 0.0026		*P*-value = 0.0561	
Field	Center	0.006a	(0.000–0.012)	0.002a	(0.000–0.004)	0	–	0	–
	Margin	0.008a	(0.002–0.014)	0.003a	(0.000–0.005)	0	–	0	–
		*P*-value = 0.6938		*P*-value = 0.5668		–		–	

Values sharing the same letter do not differ significantly at α ≤ 0.05.

Following application of Platinum 75SG to the soil in the field, no sample date by leaf section interaction was observed for thiamethoxam (*F*_7,191.1_ = 0.08; *P* = 0.9992) or for clothianidin (*F*_7,189.9_ = 0.02; *P* = 1.000). Furthermore, no significant difference in titer was observed between leaf margin and leaf center for thiamethoxam (*F*_1,191.1_ = 0.16; *P* = 0.6938; Table 3) or for clothianidin (*F*_1,189.9_ = 0.33; *P* = 0.5668; Table 3). In contrast to observations from the greenhouse, TZMU and TZNG were not detected in the field.

Following the application of Belay 2.13SC in the greenhouse, no sample date by leaf section interaction was observed for clothianidin (*F*_9,1_ = 3.49; *P* = 0.3944) and no significant difference in leaf section was observed for clothianidin (*F*_1,6.418_ = 2.28; *P* = 0.1785; Table 4). A sample date by leaf section interaction was observed for TZMU (*F*_11,12.9_ = 3.01; *P* = 0.0315), yet the order between leaf sections remained constant over time with the exception of 5 wk following application. However, a significant difference in TZMU titer was observed between leaf margin and leaf center (*F*_1,1.29_ = 81.54; *P* = 0.0402; Table 4) where the leaf margin had higher TZMU concentrations than the leaf center. Likewise, a sample date by leaf section interaction was observed for TZNG (*F*_12,3.657_ = 8.12; *P* = 0.0356), but the order between leaf section concentration remained constant across all sample dates. Furthermore, a significant difference in TZNG titer was observed (*F*_1,5.897_ = 102.05; *P* < 0.0001; Table 4) where the leaf margin had a higher TZNG titer than the leaf center. When Belay 2.13SC was applied to the soil in the field, no sample date by leaf section interaction was observed for clothianidin (*F*_7,146_ = 0.64; *P* = 0.7256) or for TZNG (*F*_7,145.9_ = 1.21; *P* = 0.3019). No difference was detected between leaf margin and leaf center for clothianidin (*F*_1,146_ = 3.37; *P* = 0.0685; Table 4), yet higher levels of TZNG occurred in the leaf margin than the leaf center (*F*_1,145.9_ = 10.05; *P* = 0.0019; Table 4). In contrast to that observed in the greenhouse experiment, TZMU was not detected in the field.

**Table 4.** Chemical titer (ppm) in citrus leaf tissue across two leaf sections following application of Belay 2.13SC (1.27 ml per tree) to the soil in the greenhouse and in the field

Study	Leaf section	Clothianidin		TZMU		TZNG	
		Mean	95% CI	Mean	95% CI	Mean	95% CI
Greenhouse	Center	38.425a	(27.890–48.960)	0.340a	(0.191–0.488)	6.595a	(5.536–7.653)
	Margin	48.306a	(37.771–58.841)	0.522b	(0.374–0.669)	9.649b	(8.592–10.707)
		*P*-value = 0.1785		*P*-value = 0.0402		*P*-value < 0.0001	
Field	Center	0.138a	(0.115–0.160)	0	–	0.033a	(0.020–0.046)
	Margin	0.159a	(0.137–0.182)	0	–	0.055b	(0.042–0.068)
		*P*-value = 0.0685		–		*P*-value = 0.0019	

Values sharing the same letter do not differ significantly at α ≤ 0.05.

### Temporal Expression of Neonicotinoids

When Admire Pro was applied to the soil in the greenhouse, a significant effect of sample date was observed for imidacloprid (*F*_12,78_ = 7.4; *P* < 0.0001; Table 5), 5-OH (*F*_12,17.25_ = 10.71; *P* < 0.0001; Table 5), and olefin (*F*_12,18.4_ = 11.6; *P* < 0.0001; Table 5). The titer of each analyte peaked at 5 wk following application and persisted through 13 wk following application. The highest mean imidacloprid titer observed was 192.060 ppm, whereas the highest mean titer for 5-OH and olefin was 33.673 ppm and 8.134 ppm, respectively. Following the application of Admire Pro in the field, a location by sample date interaction was observed for imidacloprid (*F*_9,935_ = 18.54; *P* < 0.0001), and imidacloprid titer was affected by sample date (*F*_9,935_ = 48.84; *P* < 0.0001; Table 6). The highest mean imidacloprid titer was observed 1 wk following application at the flatwoods location (1.052 ppm) and just before application at the central ridge location (1.246 ppm) (Table 6). Low levels (<0.090 ppm) of imidacloprid were detected up to 8 wk following application at the flatwoods location and 10 wk following application at the central ridge location. A location by sample date interaction was also observed for 5-OH following the application of Admire Pro to the soil in the field (*F*_9,935_ = 15.26; *P* < 0.0001), and a significant effect of 5-OH titer was observed by sample date (*F*_9,935_ = 45.85; *P* < 0.0001; Table 6). At the flatwoods location, 5-OH titer remained relatively constant up to 2 wk following application before decreasing, whereas the 5-OH titer at the central ridge location was highest prior to application and continuously decreased over time (Table 6). Furthermore, a location by sample date interaction was observed for olefin (*F*_9,935_ = 2.52; *P* = 0.0076) following the application of Admire Pro, and olefin titer was affected by sample date (*F*_9,935_ = 23.66; *P* < 0.0001; Table 6). At each location, olefin persisted for up to 6 wk following application of Admire Pro.

**Table 5.** Chemical titer (ppm) in citrus leaf tissue during the weeks following application of Admire Pro (1.48 ml per tree) to the soil in the greenhouse

Weeks following application	Imidacloprid		5-OH		Olefin	
	Mean	95% CI	Mean	95% CI	Mean	95% CI
0	00.000d	–	0cd	–	0bc	–
1	60.148cd	(00.000–128.178)	3.548c	(00.147–6.948)	0.269b	(0.137–0.402)
2	153.770ab	(85.279–222.261)	14.474abc	(10.940–18.007)	1.796b	(1.402–2.191)
3	167.060ab	(87.238–246.882)	25.178a	(20.784–29.571)	4.375ab	(3.523–5.227)
4	171.690a	(102.554–240.826)	27.001a	(21.031–32.972)	5.191ab	(4.033–6.349)
5	192.060a	(122.523–261.597)	33.673a	(25.500–41.845)	8.134a	(5.917–10.350)
6	164.640ab	(91.245–238.035)	26.616a	(19.821–33.411)	5.091ab	(3.286–6.897)
7	137.020abc	(61.172–212.868)	22.544ab	(14.249–30.838)	5.339ab	(2.891–7.786)
8	101.190abcd	(31.965–170.415)	16.796abc	(10.968–22.625)	4.896ab	(2.853–6.940)
9	102.810abcd	(33.698–171.922)	18.309ab	(11.883–24.734)	5.920ab	(2.776–9.064)
10	64.766bcd	(00.000–134.007)	9.376bc	(04.023–14.729)	2.390b	(1.642–3.138)
11	105.670abcd	(07.877–203.463)	14.260abc	(03.686–24.834)	1.956b	(0.952–2.961)
12	93.438abcd	(10.572–176.303)	18.015abc	(05.753–30.277)	6.250ab	(0.600–11.900)
13	40.810d	(00.000–108.649)	8.219bc	(03.094–13.343)	3.834ab	(1.197–6.470)
	*P*-value < 0.0001		*P*-value < 0.0001		*P*-value < 0.0001	

Values sharing the same letter do not differ significantly at α ≤ 0.05.

**Table 6.** Chemical titer (ppm) in citrus leaf tissue during the weeks following application of Admire Pro (1.48 ml per tree) to the soil in the field at two commercial Florida citrus groves

Location	Weeks following application	Imidacloprid		5-OH		Olefin	
		Mean	95% CI	Mean	95% CI	Mean	95% CI
Flatwoods	0	0.926bc	(0.777–1.075)	0.440bcd	(0.375–0.505)	0.142abc	(0.091–0.193)
	1	1.098ab	(0.989–1.207)	0.436bcd	(0.388–0.483)	0.096bcd	(0.059–0.133)
	2	1.052ab	(0.958–1.147)	0.447bc	(0.405–0.488)	0.143ab	(0.111–0.175)
	3	0.902bc	(0.812–0.992)	0.390cd	(0.351–0.429)	0.055cd	(0.024–0.085)
	4	0.539e	(0.449–0.630)	0.270ef	(0.231–0.310)	0.106abc	(0.076–0.137)
	5	0.491ef	(0.400–0.583)	0.223efg	(0.183–0.263)	0.103bcd	(0.072–0.134)
	6	0.301fg	(0.208–0.394)	0.174fgh	(0.133–0.215)	0.048cd	(0.016–0.079)
	8	0.090gh	(0.000–0.185)	0.045ij	(0.004–0.087)	0.000efg	–
	10	0.000h	–	0.000ij	–	0.000efg	–
	12	0.000h	–	0.000j	–	0.000fg	–
Central ridge	0	1.246a	(1.097–1.395)	0.606a	(0.541–0.672)	0.210a	(0.160–0.261)
	1	1.117ab	(0.988–1.246)	0.539ab	(0.482–0.595)	0.126abc	(0.082–0.169)
	2	0.885bc	(0.788–0.982)	0.434bcd	(0.391–0.476)	0.141abc	(0.109–0.174)
	3	0.784cd	(0.694–0.873)	0.393cd	(0.354–0.432)	0.014def	(0.000–0.045)
	4	0.561de	(0.470–0.653)	0.296de	(0.256–0.335)	0.096abc	(0.065–0.127)
	5	0.484ef	(0.390–0.577)	0.255efg	(0.214–0.296)	0.066bcd	(0.035–0.098)
	6	0.398ef	(0.302–0.494)	0.289de	(0.246–0.331)	0.038cde	(0.006–0.071)
	8	0.149gh	(0.036–0.262)	0.157gh	(0.108–0.207)	0.000fg	–
	10	0.006h	(0.000–0.125)	0.069hi	(0.018–0.121)	0.000g	–
	12	0.000h	–	0.000ij	–	0.000g	–
		*P*-value < 0.0001		*P*-value < 0.0001		*P*-value < 0.0001	

Values sharing the same letter do not differ significantly at α ≤ 0.05.

When Platinum 75SG was applied to the soil in the greenhouse, sample date had a significant effect on thiamethoxam titer (*F*_12,5.146_ = 7.94; *P* = 0.015; Table 7), where thiamethoxam expression was highest at 3 wk (271.140 ppm) following application. Similarly, sample date had a significant effect on clothianidin titer (*F*_12,5.068_ = 6.16; *P* = 0.0274; Table 7) and TZMU titer (*F*_12,34.62_ = 5.92; *P* < 0.0001; Table 7) following the soil application of Platinum 75SG, which also peaked at 3 wk (99.379 ppm and 3.019 ppm, respectively) following application. Although sample date significantly affected TZNG expression (*F*_12,12.65_ = 134.66; *P* < 0.0001; Table 7) following the soil application of Platinum 75SG, no clear TZNG peak was observed at a single time point; TZNG titer fluctuated over the weeks following application. In contrast to expression levels observed in the greenhouse, when Platinum 75SG (0.37g / tree) was applied to the soil in the field, limited quantifiable thiamethoxam titers, or resulting metabolite titers were detected in citrus leaf tissues, thus residue analyses for these citrus leaf tissues were ceased.

**Table 7.** Chemical titer (ppm) in citrus leaf tissue during the weeks following application of Platinum 75SG (0.37g per tree) to the soil in the greenhouse

Weeks following application	Thiamethoxam		Clothianidin		TZMU		TZNG	
	Mean	95% CI	Mean	95% CI	Mean	95% CI	Mean	95% CI
0	00.000cd	–	00.000cd	–	0.000b	–	0.000bc	–
1	69.801abc	(26.922–112.679)	12.293c	(00.000–27.877)	0.000b	–	0.943b	(0.762–1.123)
2	240.070ab	(152.601–27.539)	66.470abc	(36.160–96.780)	1.039b	(0.149–1.928)	4.028ab	(1.873–6.182)
3	271.140a	(180.863–361.417)	99.379a	(74.223–124.534)	3.019a	(2.129–3.908)	5.358a	(5.121–5.594)
4	92.293abc	(51.673–132.912)	40.939abc	(24.869–57.008)	0.701b	(0.000–1.591)	2.503b	(2.249–2.756)
5	118.970abc	(68.429–169.511)	58.515abc	(37.710–79.320)	0.855b	(0.000–1.744)	3.830ab	(2.864–4.796)
6	86.693abc	(47.322–126.063)	51.415abc	(35.267–67.563)	0.478b	(0.000–1.367)	2.795ab	(2.233–3.357)
7	114.820abc	(61.219–168.421)	68.703ab	(46.374–91.031)	0.692b	(0.000–1.581)	5.228ab	(4.014–6.441)
8	58.246abc	(17.836–98.656)	61.495abc	(31.895–91.095)	1.025b	(0.136–1.914)	5.313ab	(3.636–6.989)
9	62.636abc	(23.276–101.997)	43.769abc	(25.739–61.799)	0.886b	(0.000–1.775)	2.690ab	(1.268–4.112)
10	57.736abc	(18.481–96.992)	39.940abc	(24.144–55.736)	0.969b	(0.079–1.858)	2.728ab	(1.664–3.791)
11	44.153bc	(00.517–87.788)	27.114bc	(11.430–42.797)	1.176b	(0.287–2.066)	3.528ab	(2.760–4.295)
12	49.399abc	(07.219–91.579)	43.935abc	(19.780–68.090)	1.011b	(0.121–1.900)	4.940ab	(1.777–8.103)
13	26.421c	(26.421–65.464)	32.336bc	(16.303–48.370)	0.453b	(0.000–1.342)	3.400ab	(2.344–4.456)
	*P*-value = 0.0150		*P*-value = 0.0274		*P*-value < 0.0001		*P*-value < 0.0001	

Values sharing the same letter do not differ significantly at α ≤ 0.05.

Following the soil application of Belay 2.13SC in the greenhouse, a significant effect was observed by sample date for clothianidin (*F*_12,12.61_ = 14.25; *P* < 0.0001; Table 8), TZMU (*F*_11,14.9_ = 3.86; *P* = 0.0087; Table 8), and TZNG (*F*_12,15.67_ = 25; *P* < 0.0001; Table 8). The maximum mean clothianidin titer (62.226 ppm) was observed at 5 wk following application. The mean TZMU titer exhibited two distinct peaks: the first (0.543 ppm) at 4 wk following application, and the second (0.833 ppm) at 11 wk following application. A continual increase was observed for TZNG through 8 wk (peak mean 11.430 ppm) after application. As observed following application of Platinum 75SG to the soil in the field, limited quantifiable analytes were observed after application of Belay 2.13SC to the soil (1.27 ml per tree) in the field; therefore, residue analyses for these citrus leaf tissues were discontinued.

**Table 8.** Chemical titer (ppm) in citrus leaf tissue during the weeks following application of Belay 2.13SC (1.27 ml per tree) to the soil in the greenhouse

Weeks following application	Clothianidin		TZMU		TZNG	
	Mean	95% CI	Mean	95% CI	Mean	95% CI
0	00.000cd	–	0.000bc	–	0.000de	–
1	12.714c	(8.009–17.418)	0.000bc	–	1.019d	(0.000–2.465)
2	39.171ab	(33.239–45.103)	0.084b	(0.000–0.332)	2.813cd	(1.313–4.312)
3	49.430ab	(42.742–56.118)	0.419ab	(0.171–0.667)	6.349bc	(4.894–7.803)
4	55.218a	(47.285–63.150)	0.543ab	(0.295–0.790)	5.903bc	(4.445–7.360)
5	62.225a	(46.816–77.634)	0.460ab	(0.212–0.708)	9.045ab	(7.551–10.539)
6	51.076ab	(42.718–59.434)	0.232b	(0.000–0.480)	9.605ab	(8.135–11.075)
7	54.576a	(43.322–65.831)	0.129b	(0.000–0.377)	10.933a	(9.361–12.504)
8	48.741ab	(39.853–57.629)	0.451ab	(0.203–0.699)	11.430a	(9.880–12.980)
9	42.701ab	(35.428–49.975)	0.564ab	(0.316–0.812)	9.848ab	(8.329–11.366)
10	36.338abc	(21.613–51.062)	0.613ab	(0.365–0.860)	7.733abc	(5.042–10.423)
11	45.959ab	(35.325–56.593)	0.833a	(0.585–1.080)	10.649a	(9.141–12.157)
12	36.048abc	(23.594–48.501)	0.545ab	(0.266–0.824)	10.267ab	(8.044–12.489)
13	29.558bc	(17.487–41.628)	0.295ab	(0.034–0.556)	9.994ab	(6.920–13.067)
	*P*-value < 0.0001		*P*-value = 0.0087		*P*-value < 0.0001	

Values sharing the same letter do not differ significantly at α ≤ 0.05.

### Spatial Distribution of Admire Pro Analytes Throughout the Tree Canopy

**Fig. 1.**
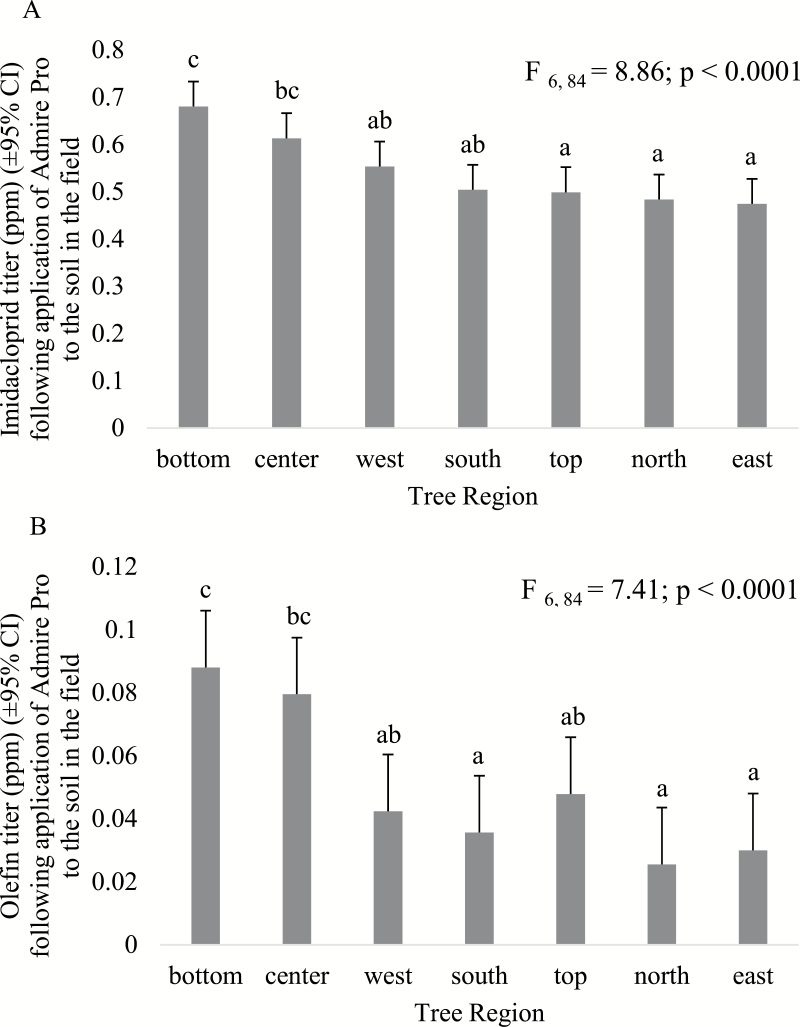
Comparison of chemical titer between seven tree regions across the 2015 and 2016 field seasons. (A) Imidacloprid titer in citrus leaf tissues resulting from soil application of Admire Pro in the field. (B) Olefin titer in citrus leaf tissues resulting from soil application of Admire Pro in the field. Bars sharing the same letter do not differ significantly at α ≤ 0.05.

**Fig. 2.**
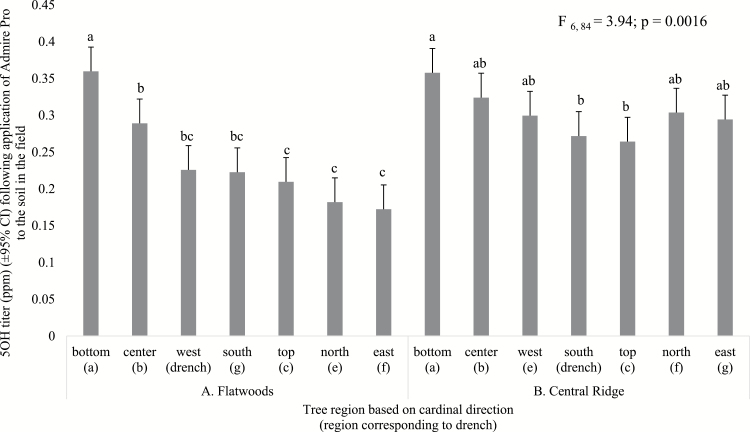
Comparison of 5-OH titer between seven tree regions at two locations across the 2015 and 2016 field seasons. (A) Tree region comparison at the flatwoods location following soil application of Admire Pro in the field. (B) Tree region comparison at the central ridge location following soil application of Admire Pro in the field. Bars sharing the same letter do not differ significantly at α ≤ 0.05.

When Admire Pro was applied to the soil in the field, we observed no sample date by tree region interaction (*F*_54,935_ = 0.61; *P* = 0.9877) and no location by tree region interaction for imidacloprid (*F*_6,84_ = 2.16; *P* = 0.0555). Tree region had a significant effect on imidacloprid titer (*F*_6,84_ = 8.86; *P* < 0.0001; Fig. 1A), in which the bottom tree region contained a significantly higher mean imidacloprid titer than the top or four cardinal side regions; no difference was observed between the spherical center region and the bottom region. Likewise, the spherical center region contained a higher mean imidacloprid titer than the top, north, or east tree regions, but was not different from the west or south tree regions. Furthermore, no difference was observed between the top tree region and the four cardinal side regions. No sample date by tree region interaction (*F*_54,935_ = 1.04; *P* = 0.3982), or location by tree region interaction (*F*_6,84_ = 0.32; *P* = 0.9249), was observed for olefin following application of Admire Pro to the soil in the field. Furthermore, a significant effect of tree region was observed for olefin (*F*_6,84_ = 7.41; *P* < 0.0001; Fig. 1B) in which the bottom tree region contained a higher mean olefin titer than the top tree region or the four cardinal side regions; no difference was observed between the bottom tree region and the spherical center region. No difference was observed in mean olefin titer between the top tree region and the four cardinal side regions, and no difference was observed between the spherical center region and the west and top tree regions. In contrast, for the analyte 5-OH, no sample date by tree region interaction (*F*_54,935_ = 1.3; *P* = 0.0777) was observed, yet a location by tree region interaction was observed (*F*_6,84_ = 3.94; *P* = 0.0016). Tree region had a significant effect on mean 5-OH titer (*F*_6,84_ = 16.65; *P* < 0.0001; Fig. 2) at the flatwoods location, where the bottom tree region contained higher 5-OH levels than all other tree regions. No difference in 5-OH titer was observed between the spherical center, west, and south tree regions, and no difference was observed between the top and four cardinal side regions. At the central ridge location, no difference was observed between 5-OH levels in the bottom, spherical center, west, north, or east tree regions, and no difference was observed between the spherical center, top, and four cardinal side tree regions.

### Effect of Leaf Maturity on Neonicotinoid Expression

After application of Admire Pro to the soil, there was no sample date by leaf maturity interaction for expression of imidacloprid (*F*_4,55_ = 0.84; *P* = 0.5053), olefin (*F*_4,55_ = 0.77; *P* = 0.5500), or 5-OH (*F*_4,55_ = 1.00; *P* = 0.4178). Moreover, no difference in titer was observed between flush shoots and mature leaves for imidacloprid (*F*_1, 7_ = 0.74; *P* = 0.4191), olefin (*F*_1,7_ = 1.95; *P* = 0.2057), or 5-OH (*F*_1,7_ = 2.55; *P* = 0.1543). Following application of Platinum 75SG to the soil, no sample date by leaf maturity interaction was observed in expression of thiamethoxam (*F*_4,55_ = 2.14; *P* = 0.0879), clothianidin (*F*_4,55_ = 1.07; *P* = 0.3823), or TZNG (*F*_4,55_ = 0.21; *P* = 0.9318). Furthermore, no difference was observed between flush shoots and mature leaves in expression of thiamethoxam (*F*_1,7_ = 2.08; *P* = 0.1929), clothianidin (*F*_1,7_ = 0.01; *P* = 0.9419), or TZNG (*F*_1,7_ = 0.04; *P* = 0.8531). No TZMU was detected following the application of Platinum 75SG to the soil. In contrast, a sample date by leaf maturity interaction was observed in clothianidin titer following application of Belay 2.13SC to the soil (*F*_4,55_ = 3.36; *P* = 0.0156). Although an interaction did occur, no difference was observed in clothianidin titer between flush shoots and mature leaves during each sample date or when sample date data were pooled (*F*_1,7_ = 5.26; *P* = 0.0554). No sample date by leaf maturity interaction was observed in TZNG titer following application of Belay 2.13SC to the soil (*F*_4,55_ = 1.28; *P* = 0.2908) and no difference was observed between flush shoots and mature leaves (*F*_1,7_ = 3.16; *P* = 0.1189). No TZMU was detected following the application of Belay 2.13SC to the soil.

## Discussion

The goal of this study was to quantify the spatial distribution and temporal expression of three currently labeled neonicotinoid insecticides in the citrus tree canopy to elucidate why trees continue to succumb to HLB infection despite intensive vector management efforts by growers. The possibility of uneven expression of neonicotinoids in citrus resulting in potential exposure of *D. citri* to sublethal dosages has been suggested previously (Boina et al. 2009, Rogers 2012). The current study was the first to use UHPLC-MS to quantify the temporal expression and spatial distribution of neonicotinoids and resulting metabolites in citrus following application to the soil. High parent material titers were observed following applications of Admire Pro (imidacloprid), Platinum 75SG (thiamethoxam), and Belay 2.13SC (clothianidin) in the greenhouse (max. mean 192 ppm imidacloprid; max. mean 240 ppm thiamethoxam; max. mean 62 ppm clothianidin). In contrast, low parent material titers (max. mean 1.246 ppm) of imidacloprid and very low titers of thiamethoxam and clothianidin (thiamethoxam max. mean 0.008; clothianidin max. mean 0.159) were detected after application in the field. Tree size and application rate are known to affect neonicotinoid expression in leaf tissues following application to the soil in the field (Langdon et al. 2018). In the present study, quantified differences in titers between greenhouse and field experiments are congruent with observations by Langdon et al. (2018), where the lowest recommended field rate applied to 2-yr old grove-planted trees yielded minimal neonicotinoid expression. Therefore, only the Admire Pro treatment in the field allowed for evaluating expression of neonicotinoids and resulting metabolites over time and space. In addition, we evaluated in-leaf distribution for all three insecticides in the field by sampling the ‘top’ tree region, which contained very low detection levels of thiamethoxam and clothianidin.

Although we did not find a difference in imidacloprid concentration between leaf sections in the greenhouse, leaf margin did contain higher levels of imidacloprid than leaf interiors following application of Admire Pro in the field. This difference was inconsistent with our findings for thiamethoxam and clothianidin following the application of Platinum 75SG and Belay 2.13SC, respectively; no difference in active ingredient expression was observed between leaf sections for either of these insecticides. Mendel et al. (2000) found low levels of ^14^C-labeled imidacloprid around leaf vascular bundles when compared with the leaf margins. Although we found a difference between leaf sections only in one case, it is possible that our excision method failed to fully account for the intricate vascular bundle–related expression patterns within citrus leaves, which may have not allowed us to detect differences. A number of the metabolites were detected at a higher concentration in the leaf margin compared with the leaf middle; however, because the concentration of each metabolite is directly dependent on the concentration of the associated parent material, it is unknown how any single metabolite may affect *D. citri* mortality. Furthermore, because radiolabeled parent material cannot be differentiated from metabolites through radiographic imaging, it is possible that patterns related to the vascular bundles observed by Mendel et al. (2000) were actually accumulations of metabolites (metabolized imidacloprid constituents) carrying the ^14^C marker instead of accumulations of the parent material, imidacloprid. Nevertheless, inconsistent neonicotinoid expression within a leaf remains of concern as it relates to *D. citri* management and potential expression of sublethal dosages.

Quantification of systemic neonicotinoid expression over time should facilitate the following: 1) determining when a subsequent non-neonicotinoid foliar spray must be applied; 2) determining the interval required to reach peak expression levels relative to application timing; and 3) understanding the persistence of neonicotinoids in leaf tissues at sublethal levels. We allowed 7 wk to elapse from the last known soil application of Admire Pro and initiation of our field studies each season. Given the prevalence of HLB infection in FL commercial groves, we did not allow more time due to the risk of developing HLB infection in cooperator groves. Interestingly, the levels of imidacloprid and metabolites observed in the preapplication samples were not statistically different from the highest mean titer observed following our treatment application. Although lethal levels of each compound, as determined by Langdon and Rogers (2017), were observed in the present greenhouse study, sublethal levels of all analytes were detected in citrus leaf tissues in the field following insecticide application to the soil. We found a peak mean titer of 1.098 ppm imidacloprid at 1 wk following application of Admire Pro at the flatwoods location, and a peak mean titer of 1.246 ppm imidacloprid at the time of application of Admire Pro at the central ridge location. It is possible that either 1) an above-label rate was applied by the grower 7 wk prior to our experimental application or 2) trees at each location were drenched by grove workers during their 6 wk soil-application rotation. Given that trees at each location contained some imidacloprid from previous grower applications at the time of our treatment application, we were unable to definitively quantify the temporal expression pattern of this insecticide in the current study. However, we were able to determine within-leaf concentration gradients and spatial distribution within tree canopies. We found higher imidacloprid concentrations in the bottom 10% of the tree canopy compared with other canopy regions, although no statistical difference was observed between the lower canopy and the spherical center.

Neonicotinoids are highly systemic and move through the plant xylem (Elbert et al. 1991, Maienfisch et al. 2001). A common characteristic of xylem mobile herbicides applied to the soil is injury accumulation in the oldest leaves. This is in contrast to phloem mobile herbicides, which cause injury near the growing point of the plant, or in the newest leaf tissue. Triazine herbicides, which like neonicotinoid insecticides are xylem mobile, result in higher concentrations within older leaves compared with new leaves when applied to the soil (Stoller 1970). The lower tree canopy contains the oldest set of leaves and our findings are consistent with movement patterns and accumulation of known xylem mobile herbicides. We did not observe a location by tree region interaction for imidacloprid expression, which indicates that the pattern of spatial distribution was not affected by the current drench practice in FL of applying neonicotinoid to the soil on one side of the tree canopy.

Young, nonbearing trees flush more frequently than mature trees throughout the year serving as preferred host sites for gravid, adult *D. citri* (Stansly and Rogers 2006, Patt and Sétamou 2010). Although *D. citri* are more attracted to flush shoots than mature leaves, much of the leaf tissue subjected to analytical evaluation of chemical titers to date have utilized only mature leaves, largely due to the constant availability of leaves within the same cohort over a long period of time following a single application (Langdon 2017). It is important to determine neonicotinoid expression levels in leaves of varying maturity to predict the effect of systemic neonicotinoid in flush shoots on *D. citri* based on known titers in mature leaves. We found no difference in titer between flush shoots and mature leaves for any chemical evaluated following application of each of the three neonicotinoids evaluated. At the time insecticide applications were made, flush shoots had emerged and were actively growing, and the same cohort was sampled across the four postapplication sample events. Subsequent studies should evaluate the relationship between bud break timing and timing of application to the soil such that resultant titers are maximized within flush shoots. Application of neonicotinoids to the soil at 2 wk prior to bud break may result in lower expression levels in flush shoots than in mature leaves due to lower availability of insecticide at the time when flush shoots emerge. Although this and other hypotheses remain to be tested, our current results indicate that neonicotinoid expression in mature leaves did not differ from that in flush shoots (only location of *D. citri* oviposition and development) when applied after bud break.

Although we successfully mapped the distribution of three neonicotinoids and one resulting metabolite within trees, we unexpectedly observed significantly lower expression levels of all compounds tested than those required to kill *D. citri* nymphs or adults by ingestion (Langdon and Rogers 2017). The size of trees chosen in the field was appropriate for the label rate applications evaluated. It is possible that HLB infection may have affected insecticide uptake of our treatment applications; however, roots could not be unearthed and inspected for disease symptoms and trees were not tested for *C*Las infection in this study. Tree size and application rate both affect uptake and expression of thiamethoxam following application to the soil in citrus (Langdon et al. 2018). While Langdon (2017) found that 0.55 ppm of imidacloprid does not affect normal *D. citri* feeding behavior as measured by electropenetrography, salivation and ingestion behaviors were reduced at 5.5 ppm of imidacloprid. Given that a peak of ca. 1 ppm of imidacloprid was measured in leaves following field application to the soil in the current investigation, it is unlikely that any of the neonicotinoid treatments evaluated here reduced *D. citri* feeding activity. Because non-neonicotinoid foliar sprays were routinely applied by growers in our field plots, no attempt was made to correlate insect incidence or abundance with neonicotinoid titer levels.

The greatest accumulations of imidacloprid quantified here occurred in the lower portion of the tree canopy. However, even those titers were below the lethal range required by *D. citri* ingestion (Langdon and Rogers 2017). Langdon and Rogers (2017) determined that the lethal dosage for *D. citri* following contact exposure to neonicotinoid insecticides is substantially lower than that required by ingestion. To potentially maximize the effective use of neonicotinoids for *D. citri* management in citrus, we suggest investigation of neonicotinoid residues over time following foliar application. Presumably, foliar application could achieve higher acutely toxic residues following application, with a more rapid residue degradation, which may be more suitable for concurrent management of *D. citri* populations and insecticide resistance. Given the recent detection of neonicotinoid resistance field populations of *D. citri* (Langdon and Rogers 2017), future neonicotinoid use in citrus must not only focus on efficacy of vector management, but also on reducing the incidence and severity of resistance.
